# Personalia

**Published:** 1913-11-29

**Authors:** 


### Personalia.

THE fine sum of £3,850 contributed by the Hospital Saturday Movement to the Coventry and Warwickshire Hospital, as recorded in THE HOSPITAL on November 15, has been followed up by two visits to the institution, in which the new extensions and improvements were inspected by the Fund Committee. Mr. C. W. Iliffe, chairman of the hospital, received the visitors, and it is interesting to note that Mr. J. A. Rudd, the secretary, whose illness we regretfully announced some time ago, was sufficiently convalescent to be present and to assist in conducting a party round the institution. A demonstration was given by Dr. Webb Fowler in the *x*-ray department, and was much appreciated. The secretarial work of the hospital is still being performed temporarily by Mr. Bernard Franklin.

A ROYAL MEDAL to Professor Ernest Henry Starling, F.R.S., for his contributions to the advancement of physiology has been granted by the Royal Society.

DR. J. EDWARD SQUIRE, C.B., F.R.C.P., who for the last thirty years has been an assistant physician and a physician at the Mount Vernon Hospital, has been elected a consulting physician.

MR. E. FITTEN, Dewsbury, Mr. Arthur Jessop, Ossett, and Mr. J. Blackburn, Heckmondwike, have been elected trustees of the Dewsbury and District Infirmary. Mr. J. Blackburn is president of the institution.

SIR RICHARD COOPER, M.P., Miss Brace, Mr. W. Bayley, Mr. W. Blackburn, Mr. Fred. J. Cotterell, Mr. F. T. Cozens, Mr. R. E. Ledbury, Mr. G. A. Phillips, Mr. J. Venables, and Mr. E. C. Windle have been recommended by the executive committee of Walsall Hospital as new trustees of the institution.

THE newly appointed Mayor of Bath, Dr. Preston King, is an honorary physician at the Royal Mineral Water Hospital and honorary assistant physician at the Royal United Hospital. Dr. King is an M.D., B.C. of Cambridge, and M.R.C.S.Eng., and the author of "Bath Waters: A Rational Account of their Nature and Use."

MR. HARRISON BUTLER, who has been in charge of the ophthalmic department of the Coventry and Warwickshire Hospital, has been appointed to the staff of the Birmingham Eye Hospital. Mr. Butler, however, is not severing his connection with the Coventry Hospital if he can count on the assistance of Dr. Duncan Davidson, one of the anæsthetists who is now specialising in ophthalmic work.

MR. LESLIE SCOTT, K.C., M.P., has been asked to accept the chairmanship of the Central Association for the Care of the Mentally Defective, now being formed, which proposes to co-ordinate all the voluntary agencies and all the statutory authorities concerned with the administration of the Mental Deficiency Act, which comes into force on April 1 next.

### THE KENSINGTON AND FULHAM GENERAL HOSPITAL BALL.

WE are asked to announce that a ball on behalf of the Kensington and Fulham General Hospital, Richmond Road, Earl's Court, S.W., will be held at Princes Galleries, Piccadilly, on Friday, December 12, 1913. Tickets, which may be obtained from Mr. G. Houghton, hon. Treasurer, or from the Secretary at the hospital, and which include supper, are 15s. each. Dancing begins at 9.30 P.M.

